# Editorial: Phenylpropanoid Systems Biology and Biotechnology

**DOI:** 10.3389/fpls.2022.866164

**Published:** 2022-03-03

**Authors:** Igor Cesarino, Aymerick Eudes, Breeanna Urbanowicz, Meng Xie

**Affiliations:** ^1^Departamento de Botânica, Instituto de Biociências, Universidade de São Paulo, São Paulo, Brazil; ^2^Synthetic and Systems Biology Center, InovaUSP, São Paulo, Brazil; ^3^Lawrence Berkeley National Laboratory, Environmental Genomics and Systems Biology Division, Berkeley, CA, United States; ^4^Department of Biochemistry and Molecular Biology, University of Georgia, Athens, GA, United States; ^5^Biology Department, Brookhaven National Laboratory, Upton, NY, United States

**Keywords:** phenylpropanoids, systems biology, biotechnology, omics, phenolics

Phenylpropanoids are specialized metabolites involved in several aspects of plant growth and development and in the responses of plants to environmental stimuli. These compounds are synthesized from key intermediates of the shikimate pathway, which are structurally modified by the combined activities of lyases, transferases, ligases, reductases and oxygenases, resulting in the organ- and developmental-specific synthesis and accumulation of diverse metabolites (Vogt, 2010). The phenylpropanoid pathway provides the building blocks for lignin, suberin, and condensed tannins that play a role in structural support and mechanical strength. Lignin is a major contributor to feedstock recalcitrance and negatively affects the conversion of plant biomass into downstream products in biorefineries (Liu et al., 2021). Further, this pathway is key for the production of anthocyanins for organ pigmentation, flavonols and flavones for UV protection, various flavonoids and isoflavonoids for plant-microbe interactions, and antimicrobial phytoalexins for protection against pathogens (Deng and Lu, 2017). In addition to their biological functions *in planta*, phenylpropanoids are economically important metabolites. They constitute important components in the human diet, acting as nutraceutical compounds with antioxidant, chemopreventive, antimitotic, neuroprotective, cardioprotective, and anti-inflammatory activities. Several phenylpropanoids are considered high-value biochemicals employed in the production of fragrances, pharmaceuticals and biopolymers (Lin and Eudes, 2020).

Systems biology approaches have enabled the characterization of numerous aspects of the phenylpropanoid metabolism in different plant species, including genes, enzymes and metabolites involved in the different branches of the pathway and how these branches are regulated. However, there is still much to be explored and determined in terms of regulation, *de novo* biosynthesis, transport, compartmentation, and polymerization of phenylpropanoids. In addition to their complex biosynthetic machinery and extensive chemical diversity, the synthesis and accumulation of metabolites is also largely dependent on the tissue type, developmental stage, plant species, or might be triggered in response to specific environmental conditions (Tohge et al., 2013). Advances in “omics” technologies provide a timely opportunity to further characterize even subtle changes in the levels of transcripts, enzymes and metabolites, and thus to provide a comprehensive systems view of phenylpropanoid metabolism throughout plant development and during stress responses. Furthermore, phenylpropanoid bioengineering holds promise to generate more resilient and nutritious crops, to maximize our arsenal of useful biomolecules, and to re-design high-yield and sustainable bioenergy feedstocks by means of biotechnology.

This Research Topic aimed to gather recent findings in all aspects of phenylpropanoid metabolism gained by means of systems biology approaches and the utilization of biotechnology to exploit the economic, medicinal and nutraceutical potential of phenylpropanoids. The topic is organized into four sections: (1) structural, molecular and computational approaches toward unraveling the biosynthetic pathways involved in synthesis of diverse phenylpropanoid-derived metabolites; (2) discovery of genes and/or gene networks involved in distinct aspects of phenylpropanoid metabolism *via* omics technology; (3) functional characterization of genes involved in the phenylpropanoid metabolism and its coordination with physiological processes; and (4) biotechnological approaches to exploit the economic, medicinal, and nutraceutical potential of phenylpropanoids.

## Structural, Molecular and Computational Approaches Toward Unraveling the Biosynthetic Pathways Involved in Synthesis Of Diverse Phenylpropanoid-Derived Metabolites

The chemical diversity of phenylpropanoids results from the modification and amplification of a set of core structures derived from the shikimate pathway. A vast array of regulatory proteins, biosynthetic enzymes, oxidases and other genes are recruited to produce the various classes of phenolic metabolites. Additionally, many phenylpropanoids are specific to just one or a few plant species, underscoring the complexity of phenylpropanoid biosynthesis and the need for comprehensive characterization studies in diverse species that expand our knowledge base beyond traditional model plant and crop species. Structural, molecular and computational approaches have been applied to identify genes, enzymes and metabolites involved in the biosynthesis of phenylpropanoids in different plants. Delli-Ponti et al. (in this volume) have reviewed how gene expression and co-expression networks can be used as tools to uncover specialized metabolism biosynthetic pathways. Also using a computational approach, Elder et al. (in this volume) have applied density functional theory (DFT) calculations to evaluate the thermodynamics of coupling modes and subsequent rearomatization reactions between coniferyl alcohol and hydroxystilbene glucosides, which has been detected as a natural monomer in the bark lignin of Norway spruce.

The biosynthesis of phenylpropanoids is often triggered by environmental stimuli. To this end, the effect of chilling treatment on the accumulation of phenylpropanoids and on antioxidant activity in seedlings of two rice varieties (contrasting for chilling tolerance) was studied by Du et al. (in this volume). Lignin is a phenolic polymer important for plant growth and development but it is also considered a major bottleneck to the efficient conversion of plant biomass into downstream products. Rosado et al. (in this volume) have reported an in-depth characterization of the structural characteristics of lignins present in rice husks and straw, which are agricultural by-products that can be used to produce chemicals and materials in biorefineries. To identify the timing and key parameters of cell wall recalcitrance across different switchgrass genotypes, Saha et al. (in this volume) measured cell wall composition and phenylpropanoid/lignin biosynthesis gene expression in three switchgrass genotypes representing lowland and upland ecotypes. Yao et al. (in this volume) reviewed recent progress in defining the lignin biosynthetic pathway in lycophytes, monilophytes, gymnosperms, and angiosperms, and integrated new insights on major transcriptional regulators. In another study with evolutionary implications, Rencoret et al. (in this volume) structurally characterized the lignin-like fractions isolated from several ancestral plants, including those from moss, lycophyte, horsetail, fern, cycad, and gnetophyte species. Blaschek and Pesquet (in this volume) provided an overview of the differences and similarities in the structures, reaction mechanisms, substrate specificities, and functional roles between phenoloxidases. Because grasses are able to synthesize phenylpropanoids from either phenylalanine (Phe) or tyrosine (Tyr), Simpson et al. (in this volume) employed ^13^C isotopic-labeled precursors and mass spectrometry-based metabolomics to determine the downstream metabolites derived exclusively from Phe and Tyr in sorghum. Several phenylpropanoids show bioactivity that might influence plant growth and development or might be beneficial for human health. El Houari et al. (in this volume) reviewed reports describing altered accumulation of bioactive phenylpropanoids (or phenylpropanoid-derived metabolites) as the causal factor for observed phenotypes of lignin mutants in Arabidopsis. Cappellini et al. (in this volume) reviewed the recent progress in understanding the anthocyanin biosynthetic pathway in plants, with special emphasis on the differences in molecular mechanisms between monocot and dicot plants, and discuss the biological activities of anthocyanins as beneficial components of the human diet. Similar to anthocyanins, tannins form another group of phenolic compounds with beneficial effects on human health. Wang et al. (in this volume) performed a genome-wide analysis of the tannase gene family to identify candidate genes responsible for tannin metabolism in three nut tree species in the Juglandaceae family: walnut, pecan, and Chinese hickory.

## Discovery of Genes and/or Gene Networks Involved in Distinct Aspects of Phenylpropanoid Metabolism *Via* Omics Technology

The identification of genes and transcriptional networks responsible for specific accumulation patterns of phenylpropanoids during a physiological development process or a stress response is essential to elucidate and harness the fine regulatory mechanisms involved in these patterns. Recent advancements in omics technologies enable integrated approaches to unravel these mechanisms at the transcriptomic, proteomic, and metabolomic levels. These studies provide platforms to guide future research on improving crops for human health and wellness. Tang et al. (in this volume) leveraged single-molecule real-time sequencing technology to elucidate flavonoid synthetic pathways in blueberries. Their transcriptome analyses led to the discovery of a R2R3 MYB transcription factor that can positively regulate anthocyanin synthesis in fruits. 5-aminolevulinic acid (ALA) is a plant growth regulator that induces fruit coloration and thereby finds potential applications in modern fruit production. A transcriptome study by Zheng et al. (this volume) identified the differentially expressed genes associated with ALA-induced anthocyanin accumulation in apple, including two R2R3-MYB transcription factors involved in flavonoid accumulation. A study by Aničić et al. (this volume) investigated flavonoid metabolism during fruit development in rockrose, a traditional medicinal plant rich in bioactive phenylpropanoids, using comparative metabolomic and transcriptomic approaches. This work highlights correlations between expression patterns of biosynthetic genes and the content of proanthocyanidins. Phenolic compounds are modulated by biotic and abiotic stresses, and a study by Laoué et al. (in this volume) used quantitative trait locus (QTL) mapping and RNA-Seq to explore the complex polygenic network underlying the constitutive production of specific stilbenoids, flavonoids, and lignans in white spruce. Understanding the formation of secondary cell walls (SCWs) and their lignification has important agro-industrial applications. Hixson et al. (this volume), by undertaking an integrated analysis of the metabolome, transcriptome, and proteome of Arabidopsis lines mutated in arogenate dehydratase genes, exposed the involvement of novel proteins and additional post-transcriptional and translational processes that govern phenylpropanoid/lignin biosynthesis. As a proxy to study SCW formation in the bioenergy crop sugarcane, Simões et al. (this volume) established a lignifying cell culture system that they probed with transcriptomic and metabolomic analyses to illuminate the molecular mechanisms involved in this differentiation process, leading to the discovery of regulatory modules that control SCW deposition.

## Functional Characterization of Genes Involved in the Phenylpropanoid Metabolism and Its Coordination With Physiological Processes

The phenylpropanoid pathway in plants plays a major role in the synthesis of a wide variety of secondary metabolites. Metabolites originating from this pathway are frequently involved in plant structure or chemical signaling and defense, including flavonoids, lignins, hydroxycinnamic esters, flavonoids, anthocyanins and tannins. Dietary flavonoids, anthocyanins, proanthocyanidins, hydroxycinnamoyl acid amides and lignans are bioactive compounds that have been shown to exhibit multiple health promoting and antioxidant activities. Lignans are plant secondary metabolites composed of a core scaffold that is formed by two or more phenylpropanoid units that can adopt a spectrum of different structural forms. Chen et al. (in this issue) identified two non-selective uridine diphosphate (UDP) glycosyltransferases (UGTs) from *Isatis indigotica* Fort. that catalyze the addition of a sugar molecule onto several structurally diverse lignin acceptor substrates. Shi et al. (in this issue) sought to explore the transcriptional regulatory mechanisms of anthocyanin and proanthocyanidin biosynthesis in Chinese bayberry, of which the fruit is considered an important dietary source of natural antioxidants. They identified a *MrMYB6* gene that is highly upregulated during the latter stages of fruit development and determined it is a negative regulator of anthocyanin and proanthocyanidins through formation of a complex with two transcription factors, bHLH and WD40. Busche et al. (in this issue) carried out a study five 2-oxoglutarate-dependent dioxygenases involved in the formation of the flavonoid aglycon in banana (*Musa*): flavanone 3-hydroxylase, flavonol synthase and anthocyanidin synthase. Biochemical analysis of several recombinant candidate proteins showed that *Musa*F3H1 and *Musa*F3H2 act as flavanone 3-hydroxylases, *Musa*FLS1 and *Musa*FLS3 both function as flavonol synthases, and *Musa*ANS has anthocyanidin synthase activity. Elucidating the activity of these genes will facilitate the development of bananas with higher nutritional value. Hydroxycinnamoyl acid amides, such as clovamide, are phenylpropanoid metabolites that play roles in protecting plants from biotic and abiotic stresses. Sullivan and Knollenberg (this issue) identified, cloned and biochemically characterized a hydroxycinnamoyl-CoA:L-DOPA hydroxycinnamoyl transferase (HDT) from red clover that is capable of synthesizing clovamide and related hydroxycinnamoyl amides *in vitro*. Characterization of this enzyme activity expands our knowledge of the poorly characterized family of BAHD hydroxycinnamoyl-CoA transferase enzymes and will aid in future studies aimed at understanding the molecular basis of substrate specificity within this important family.

Lignin is a heterogeneous phenolic polymer that is highly abundant in the secondary cell walls of all land plants and is composed of three major monolignol subunits: 4-hydroxyphenyl (H), guaiacyl (G), and syringyl (S). The monolignol building blocks of lignin are synthesized by enzymes acting in concert that catalyze sequential reactions. Lin et al. (in this issue) provide direct evidence that two key enzymes involved in monolignol biosynthesis, 4-Coumaric acid:CoA ligase (4CL) and 4-hydroxycinnamoylCoA:shikimic acid hydroxycinnamoyl transferase, form a Ptr4CL-PtrHCT complex in *Populus trichocarpa* and its formation is a potential mechanism to modulate metabolic flux during secondary cell wall synthesis. The *brown midrib* (*bmr*) phenotype found across several C4 grasses has been critical for identifying mutants compromised in lignin synthesis. Tetreault et al. (in this issue) used a combined bulk segregant analysis (BSA) and next-generation sequencing (NGA) approach to show that *bmr30* encodes a chalcone isomerase (CHI) and is involved in synthesis of the flavonoid tricin and not a monolignol. In Populus species, lignin can also be further modified by acylation with *p*-hydroxybenzoate. Zhao et al. (in this issue) used wild type (WT), lignin *p*-hydroxybenzoate deficient, and *p*-hydroxybenzoate overproduction plants to investigate the role of this modification in the response of plants to gravitropic/mechanical stress. They showed that lignin-bound *p*-hydroxybenzoate content increased during tension wood formation. This increase is correlated with a significant induction of expression of a gene encoding a BAHD family acyltransferase, namely, *p*-hydroxybenzoyl CoA: monolignol *p*-hydroxybenzoyltransferase 1 (PtrPHBMT1) whose gene product preferentially conjugates p-hydroxybenzoate to S-lignin monomer sinapyl alcohol.

## Biotechnological Approaches to Exploit the Economic, Medicinal, and Nutraceutical Potential of Phenylpropanoids

Plant phenylpropanoids and their derivatives are essential for plant growth, stress responses, and health benefits for humans. A comprehensive understanding of the biosynthetic mechanisms and transcriptional regulatory network(s) of phenylpropanoid metabolism in various plant species is central for developing biotechnological approaches to produce economically desirable traits and products. Additionally, advancements in synthetic biology and biosensor technology illuminate the potential of real-time control of phenylpropanoid metabolism in the future. Ferreira and Antunes (this volume) reviewed current progress on synthetic biology and highlighted the application of biosensors for re-engineering and autonomously controlling plant phenylpropanoid metabolism. Lam et al. (this volume) reviewed the understanding and bioengineering of the biosynthesis of tricin, a type of plant flavonoid that is an essential plant defense chemical and a promising nutraceutical. Sullivan et al. (this volume) established a *de novo* hydroxycinnamoyl-malate ester biosynthetic pathway in alfalfa *via* heterologous expression of a red clover gene and enhanced alfalfa post-harvest protein protection. A transcriptomic study of transgenic tomato plants by Zhao et al. (this volume) defined a GATA transcription factor mediating the co-regulation of drought stress response and phenylpropanoid biosynthesis. Genetic, biochemical and physiological studies from Lee et al. (this volume) found that Arabidopsis needs optimal anthocyanin content for better growth under high nitrate and high salt conditions. A study by Roldan et al. (this volume) using transgenic white clover with high levels of foliar condensed tannins discovered that condensed tannins bind to forage proteins to reduce anthropogenic greenhouse gas emission. Huber et al. (this volume) chemoenzymatically synthesized a series of new phenylpropanoid derivatives and studied their structures and biological effects. Using qualitative and quantitative phytochemical analyses, Gampe et al. (this volume) demonstrated that *Ononis* hairy root cultures produce isoflavonoids with less chemical divergence and in higher quantity, suggesting a promising system for large-scale isoflavonoid production.

Systems biology and biotechnology have largely contributed to enhance our understanding on the molecular mechanisms underlying the biosynthesis of phenylpropanoids in plants, as well as to manipulate the phenylpropanoid metabolism to exploit its economic, medicinal and nutraceutical potential. Articles in this volume further contribute to these goals, covering different aspects and branches of the pathway. Novel insights and exciting biotechnological strategies involving the phenylpropanoid pathway are expected in the years to come.

## Author Contributions

All authors listed have made a substantial, direct, and intellectual contribution to the work and approved it for publication.

## Funding

IC is indebted to Fundação de Amparo à Pesquisa do Estado de São Paulo (FAPESP) for the Research Grant No. 2019/25587-0 and to Conselho Nacional de Desenvolvimento Científico e Tecnológico (CNPq) for the research fellowship 302927/2018-2. MX is supported by the U.S. Department of Energy, Office of Science, Office of Biological and Environmental Research, as part of the Quantitative Plant Science Initiative at Brookhaven National Laboratory. BU is supported by the Center for Bioenergy Innovation (CBI), a U.S. Department of Energy Bioenergy Research Center supported by the Office of Biological, Environmental Research in the DOE Office of Science.

## Conflict of Interest

The authors declare that the research was conducted in the absence of any commercial or financial relationships that could be construed as a potential conflict of interest.

## Publisher's Note

All claims expressed in this article are solely those of the authors and do not necessarily represent those of their affiliated organizations, or those of the publisher, the editors and the reviewers. Any product that may be evaluated in this article, or claim that may be made by its manufacturer, is not guaranteed or endorsed by the publisher.

## References

[B1] Deng Y. Lu S. (2017). Biosynthesis and regulation of phenylpropanoids in plants. Crit. Rev. Plant Sci. 36, 257–290. 10.1080/07352689.2017.1402852

[B2] Lin C.-Y. Eudes A. (2020). Strategies for the production of biochemicals in bioenergy crops. Biotechnol. Biofuels 13, 71. 10.1186/s13068-020-01707-x 32318116 PMC7158082

[B3] Liu Y. Cruz-Morales P. Zargar A. Belcher M. S. Pang B. Englund E.. (2021). Biofuels for a sustainable future. Cell 184, 1636–1647. 10.1016/j.cell.2021.01.052 33639085

[B4] Tohge T. Watanabe M. Hoefgen R. Fernie A. R. (2013). The evolution of phenylpropanoid metabolism in the green lineage. Crit. Rev. Biochem. Mol. Biol. 48, 123–152. 10.3109/10409238.2012.758083 23350798

[B5] Vogt T. (2010). Phenylpropanoid biosynthesis. Mol. Plant 3, 2–20. 10.1093/mp/ssp106 20035037

